# Touch-based interventions, biofield therapies, and hypnosis for pain management: a scoping review of complementary approaches in physiotherapy

**DOI:** 10.3389/fpain.2026.1869699

**Published:** 2026-08-06

**Authors:** Francesco Bonanno, Clemente Cedro, Angelo Alito, Demetrio Milardi, Vincenzo Rizzo, Carmela Mento, Clara Lombardo

**Affiliations:** 1 Department of Clinical and Experimental Medicine, University of Messina, Messina, Italy; 2 Department of Biomedical, Dental Sciences and Morphological and Functional Imaging, University of Messina, Messina, Italy; 3 Brain Mapping Lab, Department of Biomedical, Dental Sciences and Morphological and Functional Imaging, University of Messina, Messina, Italy; 4 Department of “Scienze della Salute”, University of “Magna Graecia”, Catanzaro, Italy

**Keywords:** biofield therapies, complementary therapies, hypnosis, pain management, rehabilitation

## Abstract

**Background:**

Pain is a complex and multidimensional experience shaped by biological, psychological, and contextual factors. Complementary approaches, including hypnosis-based interventions, touch-based interventions, and biofield therapies, are increasingly explored in physiotherapy and rehabilitation as non-pharmacological strategies for pain management.

**Objective:**

This scoping review aimed to synthesize current evidence on the clinical applications, reported outcomes, and proposed mechanisms of hypnosis-based interventions, touch-based interventions, and biofield therapies in the management of pain and related outcomes.

**Methods:**

The review followed PRISMA-ScR guidelines and included randomized controlled trials (RCTs) and non-RCTs published in English between 2004 and December 2025. Databases searched were PubMed, Scopus, and Cochrane Library. Fourteen studies met the inclusion criteria, involving 1,001 patients with conditions such as fibromyalgia, chronic widespread pain, temporomandibular disorders, knee osteoarthritis, and chronic back pain.

**Results:**

Overall, hypnotherapy was associated with consistent reductions in pain intensity and improvements in psychological outcomes, including anxiety, depression, catastrophizing, and sleep quality. Several studies reported that these improvements were maintained at medium- and long-term follow-ups. Touch-based interventions and biofield therapies were associated with potential improvements in pain-related outcomes and, in some studies, physical function. Some combined approaches integrating hypnosis-based interventions, touch-based interventions, or biofield therapies with conventional treatments were associated with greater improvements than standard care alone.

**Conclusions:**

Despite encouraging findings, methodological heterogeneity and variable study quality limit definitive conclusions. Hypnosis-based interventions, touch-based interventions, and biofield therapies may represent complementary approaches to pain management and rehabilitation; however, the available safety and effectiveness evidence remains preliminary.

## 1 Introduction

Therapeutic Touch (TT) is a biofield therapy within Complementary and Alternative Medicine (CAM) that aims to restore balance in the purported human biofield. Gentle Touch Therapy (GTT), Healing Touch (HT), Reiki, and Laying on of Hands (LooH) represent related but distinct interventions and should not be considered synonymous with TT (1). CAM encompasses a broad range of health practices that lie outside the scope of conventional medicine. TT was first introduced in 1972 by Professor Dolores Krieger and pranic healer Dora Kunz at New York University (2). Their method drew upon principles originally proposed by 18th-century Austrian physician Franz Anton Mesmer, who theorized that a transferable “vital energy” flows through all living beings (3).

According to this framework, imbalances in an individual's bioenergetic field can manifest as illness, which trained practitioners attempt to correct by moving their hands near the patient's body without physical contact (4). The central premise of TT is the existence of a massless human energy field that “extends beyond the distinguishable mass that we perceive as human” (5). This belief is closely linked to vitalist theory, which posits that living organisms differ from non-living matter because they are governed by unique energetic principles (6).

TT is grounded in two main theoretical frameworks (1): Field theory, which suggests that all organisms are interconnected through a shared energy field (7), and (2) Rogers' Science of Unitary Human Beings, which conceptualizes the universe as an open and dynamic system (5).

The U.S. National Center for Complementary and Integrative Health (NCCIH) categorizes energy-based therapies into two groups (8): (1) Bioelectromagnetic therapies, involving the use or manipulation of electromagnetic fields (e.g., magnetotherapy), and (2) Biofield therapies, which act upon the purported energy fields surrounding the body. Biofield therapies include interventions such as TT, Reiki, and HT, although their protocols differ in whether and how direct physical contact is used. GTT and other manual approaches involving direct tactile interaction are discussed separately as touch-based interventions according to the protocols reported in the included studies (8). For the purposes of this review, the included interventions were organized into three conceptually distinct categories: hypnosis-based interventions, touch-based interventions, and biofield therapies. Hypnosis-based interventions involve focused attention and therapeutic suggestion. Touch-based interventions involve direct tactile or manual interaction with the patient. Biofield therapies are based on the proposed modulation of a human biofield and may be delivered either with or without direct physical contact. Although some interventions may have overlapping features, these categories are not treated as interchangeable. Each intervention is therefore described according to the protocol and terminology used in the original study.

Although Healing Touch and Reiki are generally classified as biofield therapies, some treatment protocols involve direct physical contact. In these cases, their clinical effects may also be partly explained by mechanisms related to affective touch, in addition to the proposed biofield rationale. Gentle, low-pressure stimulation activates C-tactile afferents, which encode the emotional aspect of touch and project to limbic structures involved in pain modulation (9, 10). This pathway shares innervation with vagal afferents that are involved in reducing heart rate and blood pressure during touch-based interventions (11), providing a neuroscientifically grounded alternative to the biofield construct. However, none of the included studies tested this mechanism directly, and its relevance to no-touch TT specifically remains speculative rather than established.

While TT focuses on energetic and tactile engagement with the body's biofield, hypnosis offers a different yet complementary approach to non-pharmacological pain management. It can be seen as “*a waking state of awareness (or consciousness), in which a person's attention is detached from his or her immediate environment and is absorbed by inner experiences such as feelings, cognition and imagery*” (12). Though its historical roots trace back to ancient Egyptian and Roman times (13), hypnosis gained scientific credibility in the 20th century through the work of psychiatrist Milton Hyland Erickson.

His technique, called Ericksonian hypnosis (14), allows communication with the patient's unconscious, inducing a state of hypnotic trance using persuasive and poetic language. In clinical terms hypnosis is “*a procedure in which a practitioner helps to change sensations, perceptions, thoughts or behaviors relating to an experience lived by the patient*” (15). This method is based on a state of focused attention and concentration.

Several neurophysiological studies have investigated the mechanisms underlying hypnosis (16). Ulrich et al. (17) demonstrated a 16% increase in regional cerebral blood flow during hypnosis, with specific involvement of the right occipital and temporal regions. Maquet et al. (18), on the other hand, explored the brain mechanisms “at rest” and concluded that the hypnotic state is related to the metabolic activation of cortical areas such as the occipital, parietal, precentral, premotor, ventrolateral, left prefrontal and anterior cingulate cortices (ACC).

This widespread pattern resembles the activation observed during mental imagery and the retrieval of autobiographical memories. This supports the idea that hypnosis is a state involving the broad reorganisation of networks responsible for attention and the regulation of consciousness, rather than activity confined to a single region (19). Notably, Maquet et al. also reported a reduction in pain perception of around 50% under hypnosis. This early link between this network-level shift and hypnotic analgesia was later corroborated by subsequent neuroimaging work on the cingulate cortex, which is discussed below.

One of the most widely studied clinical applications of hypnosis is pain modulation. In clinical contexts, hypnosedation is the use of hypnosis alongside local anesthesia. Many neuroimaging studies have investigated brain mechanisms that allow hypnosis to modulate pain perception by inhibiting the activation of the ACC, thalamus, brainstem, and other subcortical structures (20, 21).

These mechanisms may be of clinical relevance for pain conditions in which standard pharmacological approaches have limited efficacy, such as fibromyalgia, complex regional pain syndrome, and small fiber neuropathies, conditions frequently associated with chronic neuropathic or centralized pain, impaired quality of life, and substantial diagnostic and therapeutic challenges (22–25).

Functional neuroimaging suggests that under hypnosis, pain stimuli fail to activate areas typically involved in pain processing, possibly due to altered thalamic activity (26) and changes in ACC connectivity (27, 28). These findings support the hypothesis that hypnosis facilitates subcortical “gating” mechanisms that reduce both the perception and emotional response to pain (21).

Similarly, TT contributes to pain modulation through both physiological and communicative pathways. Pain Neuroscience Education (PNE) and the use of verbal, paraverbal, and nonverbal communication allow clinicians to reframe the patient's understanding of pain as a reversible and dynamic phenomenon (29, 30). While the biological dimension of pain may be addressed through exercise and load adaptation (31), the psychosocial dimension can be influenced by contextual cues such as language, touch, and therapeutic presence (32). In this sense, every gesture or word by the physiotherapist becomes a communicative act that may alter the patient's perception and experience of pain. From a biopsychosocial perspective, hypnosis represents a conceptual bridge between psychological processes and rehabilitation practice, as it acts directly on cognitive, attentional, and emotional mechanisms that shape the experience of pain (33). Hypnosis induces a state of focused attention and increased responsiveness to therapeutic suggestions, allowing modulation of the sensory and affective dimensions of pain through top-down regulatory pathways (16, 20). Neuroimaging studies have shown that hypnotic modulation involves cortical and subcortical structures, such as the anterior cingulate cortex and thalamus, supporting the hypothesis that pain perception can be altered through changes in attentional control and emotional appraisal (21, 26). In rehabilitation settings, this approach does not replace physiotherapeutic interventions but may enhance their effectiveness by reducing anxiety and maladaptive beliefs, while strengthening patients' sense of control and self-efficacy (32). Based on the included evidence, hypnosis may be considered a complementary clinical tool that facilitates the integration of physical treatment, therapeutic communication, and psychological meaning-making within a person-centred model of care, as reflected in several of the included studies evaluating hypnosis for chronic pain management (34–40). Some included studies evaluated additional or combined approaches, including Reiki and mindfulness (41), and progressive relaxation (34, 35). Reiki was classified as a biofield therapy, whereas mindfulness and progressive relaxation were considered distinct behavioral or attentional components. Their inclusion reflects the protocols of eligible studies and does not imply a shared mechanism across interventions.

Within this integrative framework, which acknowledges the interaction between physiological, psychological, and contextual factors in pain modulation, the present scoping review aims to summarize the available evidence on hypnosis-based interventions, touch-based interventions, and biofield therapies in pain management and rehabilitation.

The aim of this scoping review is to summarize the available literature on hypnosis-based interventions, touch-based interventions, and biofield therapies for pain management. Specifically, this review attempts to (1) summarize the physiological and psychological mechanisms proposed for hypnosis-based interventions, touch-based interventions, and biofield therapies, (2) explore the clinical use of these complementary approaches in different painful conditions, (3) analyze the methodological quality and heterogeneity of existing studies, and (4) highlight research gaps to guide future investigations and clinical guidelines. Considering the general exploratory purpose of scoping reviews, the authors aim to provide a synthesis of the current evidence through a structured literature review and to identify applicability in physiotherapy practice.

## 2 Materials and methods

### 2.1 Protocol

This scoping review on hypnosis-based interventions, touch-based interventions, and biofield therapies for pain management was conducted in accordance with the Preferred Reporting Items for Systematic reviews and Meta-Analyses extension for Scoping Reviews (PRISMA-ScR) guidelines (42). The PICOS framework was used to guide the eligibility criteria, as summarized in Table 1. This study did not involve human or animal subjects and therefore did not require ethical approval.

**Table 1:** PICOS criteria.

Criteria	Applications
Population	Patients with painful conditions
Intervention	Therapeutic Touch OR Healing Touch OR Reiki OR Gentle Touch Therapy OR Hypnosis
Comparison	Placebo or sham intervention, usual/standard care, waitlist control, or active comparators (e.g., conventional physiotherapy/kinesitherapy)
Outcome	Pain intensity and pain-related outcomes, quality of life, psychological symptoms, and functional/disability outcomes.
Study Design	RCT and non-randomized study

### 2.2 Search protocol

The authors performed a structured literature search for English articles published between January 2004 and December 2025 on the electronic databases PubMed, Scopus and Cochrane Library. This twenty-year timeframe was chosen in advance to ensure the study's relevance to current clinical and rehabilitative practices, given the significant evolution of pain management paradigms that has occurred during this period. Full database-specific search strings are reported in Table 2.

**Table 2:** Database-specific search strings.

Database	Search String
Pubmed	(“Pain”[Mesh] OR pain[tiab]) AND (“Therapeutic Touch”[Mesh] OR “therapeutic touch”tiab] OR “Healing Touch”[tiab] OR hypnosis[Mesh] OR hypnosis[tiab] OR Reiki[tiab] OR “gentle touch therapy”[tiab]) AND (“Quality of Life”[Mesh] OR “quality of life”[tiab])
Scopus	(pain AND (therapeutic touch OR hypnosis OR healing touch OR Reiki OR gentle touch therapy) AND quality of life)
Cochrane Library	(pain AND (therapeutic touch OR hypnosis OR healing touch OR Reiki OR gentle touch therapy) AND quality of life)

### 2.3 Inclusion and exclusion criteria and selection of sources of evidence

**Figure 1:**
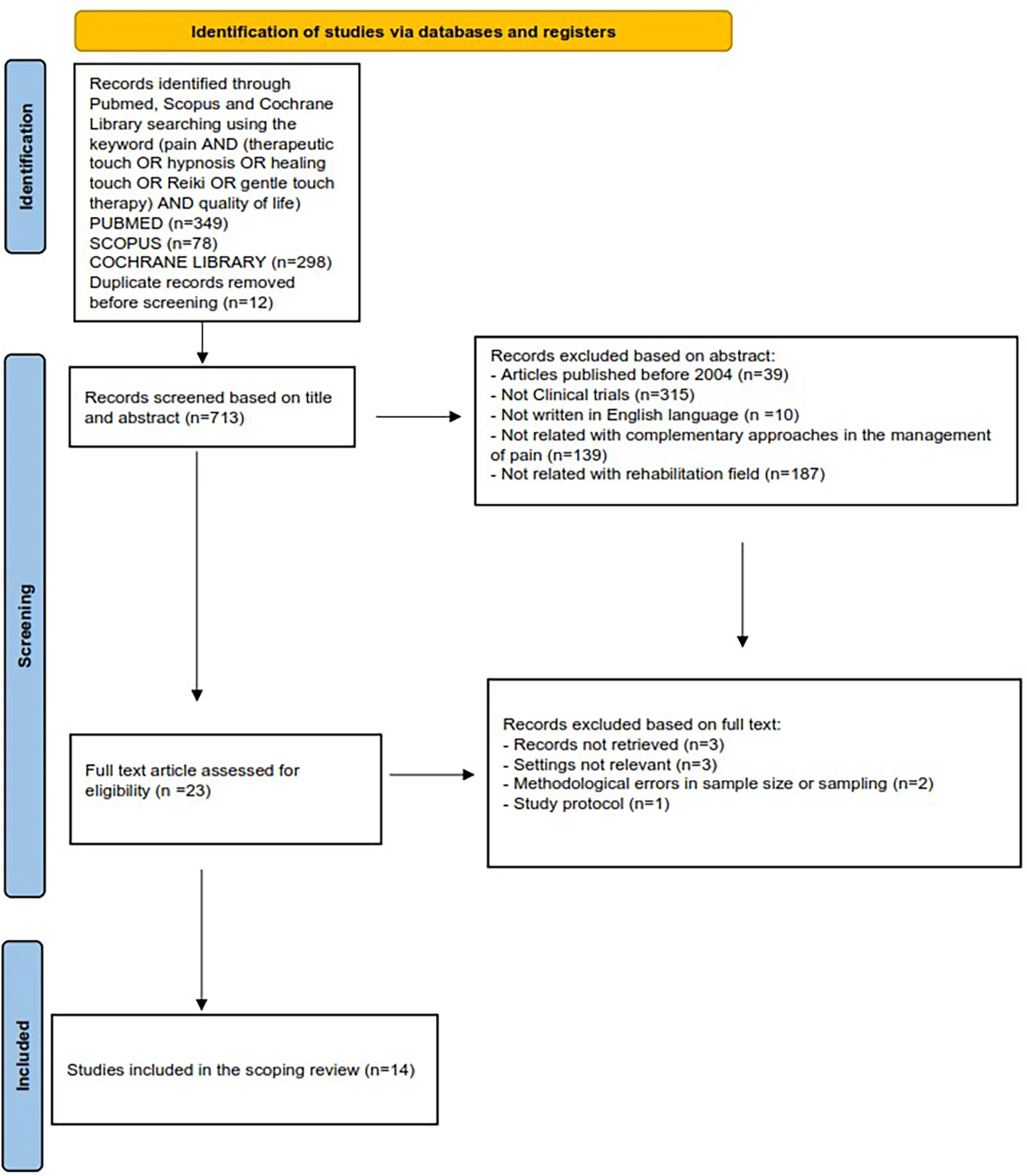
Preferred reporting items for systematic reviews and meta-analyses extension for scoping reviews (PRISMA-ScR) flowchart resuming the paper's selection process.

Grey literature and trial registries were not systematically searched, to preserve consistency of the risk-of-bias and quality appraisal (RoB 2, NOS, AHRQ) across all included evidence, which generally requires a level of methodological reporting not available in unpublished sources. PRISMA-ScR (42) does not mandate grey literature search as an obligatory element of scoping reviews. Two independent observers performed the screening and analysis separately. Firstly, articles were screened by title and abstract, using the following inclusion criteria for selection: (1) randomized controlled trials (RCTs) and non-randomized studies; (2) written in English language; (3) published in indexed journals from 2004 to 2025; and (4) dealing with hypnosis-based interventions, touch-based interventions, or biofield therapies, including hypnosis, TT, GTT, HT, Reiki, and LooH, for pain management and related quality-of-life outcomes. The exclusion criteria were (1) reviews; (2) papers written in languages other than English; and (3) data that do not concern the above-mentioned therapies and the rehabilitation field. Secondly, the full texts of the selected articles were screened with further exclusions according to the criteria described above. A PRISMA flowchart of the selection and screening method is provided in Figure 1 (Preferred Reporting Items for Systematic Reviews and Meta-analyses flowchart resuming the paper's selection process).

### 2.4 Data extraction

Authors extracted and collected the relevant data according to the Levels of Evidence (LOE) (43) into a single database: (1) study design, (2) pathology, (3) sample size and patients' features, (4) outcome measures, (5) therapeutic protocol and follow-up, (6) a summary of clinical results (Table 3), and (7) LOE (Table 4).

**Table 3:** Demographic, clinical characteristics of patients and a descriptive analysis of the interventions: data extracted from studies included.

Publication	Study design	Pathology	Score	Patients feature	Therapeutic protocol and F-up	Induction/delivery technique	Control/Comparator condition	Results	Side effects	LOE
Hanley et al. (41)	A placebo-controlled RCT	Chronic knee pain	WOMAC	157 patients (39 vs. 40 vs. 39 vs. 39)	Reiki group: 4 weekly 30-min sessions	Reiki: non-contact hand positions + brief discussion	Sham Reiki (“Feiki”: identical movements, no healing intention) + waitlist control	Both Reiki and mindfulness groups produced statistically significant improvements vs. waitlist in WOMAC symptom severity at 2-month follow-up	No treatment-related adverse effects	1
				M = 52	Feiki group (placebo)					
				F = 101	Mindfulness group: 4 weekly 30-min					
				Age 58.08 years	Waitlist group (control group)					
					F-up at baseline, 1-month, and 2-month					
Dorta et al. (38)	RCT, blindly evaluated	Fibromyalgia	PI-NRS; SF-MPQ; NRS-PU; PCS; HADS; PSQI; FIQ; WHOQOL	49 patients (24 vs.25)	Hypnosis group: 1 h hypnosis session + 8 weekly	Structured hypnosis session; technique not further specified	Attention control (neutral conversation, same session frequency)	Improved pain, sleep, mood, and QoL in hypnosis group	No adverse effects reported	1
				Mean age 45.4	F-up at 3 months					
Salgado et al. (46)	Double-blind RCT, placebo & healthy controls	Fibromyalgia	VAS; SF-MPQ; FIQ; SF-36	82 patients 64 women with fibromyalgia (32 vs.32)	GTT group performed two GTT	Micro-physiotherapy: manual palpation to identify tissue mobility restrictions, followed by gentle approximation/separation hand movements guided by a dermal map	Sham/placebo GTT (same movements, simulated approach)	Pain reduction in GTT group; no QoL improvement	Not available	1
				18 healthy subjects (control group) mean age ∼54	Sessions Evaluations up to 180 days					
Tonye-Geoffroy et al. (40)	RCT	Chronic non-cancer pain	VAS; SF-36	72 patients (34 vs. 36)	Intervention group: hypnosis + TENS session;	Hypnosis + TENS	Active comparator: TENS alone	Greater pain/QoL benefit with combined therapy	6 patients with ≥1 non-serious adverse effectrs (3/group); 4 treatment-related (pain increase n = 2, shock sensation *n* = 1, electrode intolerance *n* = 1)	1
				Mean age 49.1	F-up at 3 and 6 months					
Bicego et al. (37)	Open-label longitudinal RCT	Chronic pain	NRS; HADS; ISI; PDI; MHLC; SOPA-35; SF-36; PGIC	203 patients (45 vs.47 vs. 52 vs. 59)	Four parallel arms (7 monthly sessions, F-up at 6/12 months): psychoeducation/CBT, self-care alone, self-hypnosis + self-care (20-min hetero-hypnosis), music + self-care (20-min melody)	Hetero-hypnosis exercise (practitioner-guided)	3 active comparators to self-hypnosis/self-care: psychoeducation/CBT, self-care alone, music/self-care (no inactive/waitlist control)	All four groups improved over time (pain, insomnia, physical QoL); no significant group effect — self-hypnosis/self-care was not superior to the other arms. Benefits maintained at 6/12-month follow-up.	Not available	2
				Sex: M = 21 F = 182 Mean age 46.7 ± 8.77						
Aravena et al. (36)	RCT	Fibromyalgia	BPI-SF, BFI, CES-D, SWLS	97 patients (48 vs.49)	Hypnosis for 30 days.	Audio-recorded hypnosis (self-administered, daily)	Waitlist control; both groups continued usual pharmacological/physical/psychological treatment unchanged.	Reduced pain/fatigue; better mood/life satisfaction	Not available	1
				Sex M = 3 F = 94 Mean age 45	Follow-up at 1 and 6 months					
Zacaron et al. (45)	RCT	KOA	WOMAC; VAS: HADS	120 patients (40 vs. 40 vs. 40) Mean age 69	Kinesiotherapy + spiritual/non-spiritual LooH	Laying on of Hands, with/without spiritual component	Active comparator: kinesiotherapy alone (no LooH)	Spiritual LooH: better pain, function, anxiety outcomes	No adverse effects reported	1
					F-up at 8 & 16 weeks					
Mueller et al. (49)	Experimental Pilot Study	Chronic back pain	Quebec Back Pain Disability Scale; NPRS	29 patients (15 vs.14)	Therapeutic touch group: 4 daily sessions over 4 consecutive days	Therapeutic Touch (standard protocol)	Active comparator: standard pharmacologic pain management	Intervention group showed significant reduction in disability scale and pain scores	Not systematically assessed	2
				M = 17 F = 12 Age 59.31 years	F-up at baseline and after 4 days treatment					
Wardell et al. (47)	RCT + qualitative	Persistent pain	Verbal Descriptor Scale; PATCIE; Katz Index of Independence in Activities of Daily Living	20 patients (12 vs. 8)	Intervention group performed Healing Touch intervention 3 times per week for 30 min over 2 weeks;	Healing Touch (standard protocol)	Attention control (“presence care,” non-intervention)	Mixed results: some improved, others no benefit	None reported	2
Abrahamsen et al. (35)	RCT (Hypnosis vs. Relaxation)	TMD	NRS	40 women with myofascial TMD (20 vs. 20) Mean age 38.6 ± 10.8 yrs	Intervention group performed 4 hypnosis sessions of 1 h; Evaluation at baseline and post-treatment	Progressive relaxation + guided imagery + direct suggestions for pain modulation/coping	Active comparator: relaxation therapy (5 weeks, no therapeutic suggestions)	Intervention group: − Pain − Anxiety − awakenings + coping	No treatment-related adverse effects	1
Smith et al. (48)	Pilot RCT (single-blinded)	KOA	SF-36, WOMAC, KSS	60 patients with knee OA (28 vs.32), aged >50,	Intervention group: 2 Therapeutic Touch sessions/week for 8 weeks;	Standard Krieger-Kunz TT protocol, administered by people trained/experienced in TT	Usual care: standard physiotherapy	Significant improvement in pain and physical function	Not available	1
					F-up at 12–16 weeks					
Abrahamsen et al. (34)	RCT controlled, patient-blinded	Persistent idiopathic orofacial pain	VAS, MPQ, SCL, SF-36	41 patients (22 vs. 19)	Hypnosis group: 5 hypnosis sessions;	Progressive relaxation + guided imagery + direct suggestions for pain modulation/coping	Active comparator: relaxation-only session	Significant pain reduction in hypnosis group	Not available	2
				Sex M = 6 F = 35 Mean age 56 ± 1.9	F-up before the first treatment and at the end of each treatment					
Grøndahl et al. (39)	RCT pilot study	CWP	WHOQOL BREF; SHC; SCL-5	16 patients (8 vs. 8)	10 weekly hypnosis sessions; F-up at 1 year	Standardized, manualized protocol; induction–theme; ego-strengthening, relaxation, visualization; sessions audio-recorded for home use	Usual/routine general-practice care, no structured intervention	Lasting pain and QoL improvements	Not available	2
				M = 4						
				F = 12						
				Age 23–54 years						
Denison (50)	Pilot Clinical trial	Fibromyalgia	VAS	15 patients (10 vs.5)	Therapeutic touch group: 6 consecutive weekly treatments of therapeutic touch	Therapeutic Touch (standard protocol)	Attention control (listening to an information audio tape)	Therapeutic touch significantly decreased the experience of pain in therapeutic touch group:	Not reported	3
				Age 51.47 years	F-up at the beginning and at the end of the treatment					

BFI, Brief Fatigue Inventory; BPI-SF, Brief Pain Inventory—Short Form; CBT, Cognitive Behavioral Therapy; CES-D, Center for Epidemiologic Studies Depression Scale; CWP, Chronic Widespread Pain; FIQ, Fibromyalgia Impact Questionnaire; GTT, Gentle Touch Therapy; HADS, Hospital Anxiety and Depression Scale; ISI, Insomnia Severity Index; KSS, Knee Society Score; LOE, Level of Evidence; LooH, Laying on of Hands; MHLC, Multidimensional Health Locus of Control; MPQ-SF, McGill Pain Questionnaire—Short Form; NPRS, Numeric Pain Rating Scale; NRS, Numeric Rating Scale; NRS-PU, Numeric Rating Scale—Pain Unpleasantness; PATCIE, Perceived Ability to Cope with Trauma and Illness Events; PCS, Pain Catastrophizing Scale; PDI, Pain Disability Index; PGIC, Patient Global Impression of Change; PI-NRS, Pain Intensity—Numeric Rating Scale; RCT, Randomized Controlled Trial; SCL, Symptom Checklist; SCL-5, Short version of the SCL (5 items); SF-36, Short Form Health Survey—36 items; SHC, Subjective Health Complaints; SOPA-35, Survey of Pain Attitudes—35 items; SWLS, Satisfaction With Life Scale; TENS, Transcutaneous Electrical Nerve Stimulation; TMD, Temporomandibular Disorder(s); VAS, Visual Analogue Scale; WHOQOL (-BREF), World Health Organization Quality of Life (Short Form); WOMAC, Western Ontario and McMaster Universities Osteoarthritis Index.

**Table 4:** Levels of Evidence.

Level of Evidence (LOE)	Criteria
LOE 1: Good quality RCTs	− Blind randomization − Adequate sample size − Adequate follow-up (>80%)
LOE 2: Low quality RCTs	− No blind randomization − Inadequate sample size − Inadequate follow-up (>80%)
LOE 3: studies based on opinions, clinical practice and case series	

### 2.5 Quality assessment

Two independent researchers assessed the risk of bias, and disagreements were resolved by a discussion with a third author. The Cochrane Risk of Bias for RCT tools was used for the randomized controlled trials included in the review, while the Newcastle Ottawa Scale (NOS) was employed for the non-randomized trials. The Cochrane risk of Bias 2 (RoB 2) Tool assesses internal validity by systematically evaluating five key areas. These include randomization process, deviation from intended intervention, missing outcome data, measurement of outcomes, selection of the reported results reporting and “overall risk of bias” judgment. The results were then converted to Agency for Healthcare Research and Quality (AHRQ) standards as “good”, “fair”, or “poor” quality using the following threshold rule: studies with all RoB 2 domains rated as low risk were classified as Good; studies with at least one domain rated as some concerns were classified as Fair; and studies with at least one domain rated as high risk were classified as Poor (44).

## 3 Results

### 3.1 Study selection

A total of 725 articles were initially identified across the three databases analyzed. After removing 12 duplicate records, 713 unique records were screened by title and abstract. Of these, 690 were excluded for the following reasons: published before 2004 (*n* = 39), not clinical trials (*n* = 315), not written in English (*n* = 10), focusing on interventions outside the predefined categories of hypnosis-based interventions, touch-based interventions, and biofield therapies (*n* = 139), or not related to rehabilitation (*n* = 187). The remaining 23 articles were read in full and 9 were excluded due to the following reasons: not accessible (*n* = 3), irrelevant study setting (*n* = 3), methodological errors in sample size or sampling (*n* = 2), and study protocol with no available outcome data (*n* = 1). Ultimately, 14 articles met the inclusion criteria and were included in this review.

### 3.2 Study features

According to the inclusion criteria, all the studies included in this review were RCTs and non-randomized study published between 2004 and 2025. They involved 1,001 patients affected by various conditions associated with pain and reduced quality of life. According to the literature review, these conditions included fibromyalgia, knee osteoarthritis, chronic pain, and temporomandibular disorders. Assessments were performed at baseline (T0) and during follow-ups (T1–T2) using validated clinical tests and scales. The most used measures included the Visual Analogue Scale (VAS), the Numeric Rating Scale (NRS), and the SF-36. A full description of each study is presented in Table 3.

### 3.3 Characteristics of the included interventions and comparator conditions

Treatment modalities varied widely across studies. Control and intervention groups received different therapeutic interventions, including kinesitherapy (45); hypnotherapy (34–40); GTT (46), healing touch (47); TT (48–50) and Reiki (41). The frequency and duration of therapy sessions, as well as follow-up periods and outcome measures, differed significantly between studies.

### 3.4 Reported clinical outcome

#### 3.4.1 Hypnosis-based interventions

Dorta et al. (38) examined the multidimensional effects of hypnosis on pain, mental health, sleep quality, and overall quality of life in patients diagnosed with fibromyalgia. Participants in the intervention group received a structured hypnosis session, while the control group engaged in neutral conversation. Both groups subsequently participated in eight weekly 1-hour therapeutic sessions. Follow-up assessments were conducted at 3 months post-intervention. Outcome measures included pain intensity and unpleasantness, pain quality, pain catastrophizing, anxiety and depressive symptoms, sleep quality, fibromyalgia-related disability, and quality of life. The hypnosis group, compared to the control group, reported greater improvements across the assessed outcomes compared with the control group, a reduction of pain intensity and unpleasantness, lower levels of anxiety, depression, and catastrophizing, an improvement in sleep quality and an enhanced quality of life, including physical and psychological well-being. These effects were maintained at the 3-month follow-up, suggesting that these improvements were maintained during follow-up.

Similar results have been reported by Aravena et al. (36) evaluating the efficacy of daily audio-recorded hypnosis intervention in patients with fibromyalgia syndrome (FMS). The intervention consisted of listening to a hypnosis audio recording for 30 min daily over 30 days. The control group did not receive this intervention. Outcome measures were collected at baseline, immediately after the 30-day intervention, and at a 6-month follow-up. Key assessments included pain severity and interference, fatigue severity, depressive symptomatology, and life satisfaction. The results suggested that the hypnosis group experienced significant reductions in pain intensity and fatigue severity compared to the control group at both post-treatment and 6-month follow-up.

Abrahamsen et al. (35) reported greater improvements associated with hypnotherapy compared to relaxation training in reducing pain perceived by patients suffering from temporomandibular disorders. The intervention group underwent four weekly 1-hour sessions of clinical hypnosis, while the control group received relaxation therapy over a 5-week period. The hypnosis protocol included progressive relaxation, guided imagery to a safe and pleasant place, specific suggestions for pain modulation and improved coping. The relaxation group followed a standard protocol without therapeutic suggestions. Both groups were evaluated pre- and post-treatment, for pain intensity. The findings showed that the hypnosis group experienced significantly greater improvements compared to the relaxation group in several key areas: reduction in pain intensity, particularly during rest and jaw movement, fewer nighttime awakenings due to pain, decreased anxiety symptoms, and improved coping mechanisms. These improvements were not observed to the same degree in the relaxation group, suggesting a potential benefit of hypnosis beyond general relaxation. In another study from 2008 (34), the same authors investigated the efficacy of hypnosis in the treatment of persistent idiopathic orofacial pain (PIOP). The hypnosis protocol was the same as the article described above, as the relaxation-only session performed by the control group. Both groups were evaluated before the first session and after each subsequent treatment, using validated tools assessing pain intensity, pain quality, psychological symptoms, and quality of life. The study found that the hypnosis group showed a significant and clinically meaningful reduction in pain intensity compared to the control group. Additionally, there were marked improvements in psychological parameters, such as reduced distress levels and enhanced emotional well-being, and quality of life.

Still on hypnotherapy, Grøndahl et al. (39) explored the effectiveness of hypnosis in treating patients with chronic widespread pain (CWP) in a general practice setting. The intervention group underwent a standardized hypnosis program consisting of weekly sessions for ten consecutive weeks. The control group continued with their routine care without any structured therapeutic intervention. Outcomes were assessed using measures of quality of life, subjective health complaints, and psychological symptoms. The results revealed a significant and lasting reduction in pain levels and marked improvements in quality of life among participants in the hypnosis group compared to the control group. These benefits were sustained at a 1-year follow-up, suggesting that the reported improvements were maintained at the one-year follow-up. While the sample size was small, the study reported high adherence and positive subjective feedback from participants undergoing hypnosis, suggesting both feasibility and acceptability of the intervention in a primary care context.

Tonye-Geoffroy et al. (40) evaluated the effectiveness of combining hypnosis with transcutaneous electrical nerve stimulation (TENS) in patients suffering from chronic non-cancer pain. The intervention group participated in hypnosis sessions in conjunction with regular TENS treatment for 3 months, while the control group received TENS only. Outcome measures, including pain intensity and quality of life, were evaluated at 3 months (end of treatment) and 6 months (follow-up). Patients in the combined therapy group experienced significantly greater reductions in pain scores compared to those receiving TENS alone at the 3-month mark. Additionally, quality of life improved significantly in the combined group, particularly in physical and emotional functioning domains of the SF-36. These benefits were sustained at 6-month follow-up, suggesting that the observed improvements were maintained during follow-up.

#### 3.4.2 Touch-based interventions and biofield therapies

This subsection reports the findings separately for the specific interventions identified in the included studies, namely TT, GTT, HT, Reiki, and LooH. Regarding touch-based and biofield interventions, Salgado et al. (46) investigated the efficacy of GTT in patients with fibromyalgia. Over six months, participants in the GTT group received two sessions of Gentle Touch Therapy, each lasting 40 to 45 min, administered on Day 0 and Day 45. Outcome assessments were conducted at multiple timepoints: baseline, Day 15, Day 45, Day 60, Day 90, Day 120, and Day 180. The primary outcome was pain intensity along with secondary outcomes including fibromyalgia-related impact, quality of life, and pain quality. The patients in the GTT group reported a significant reduction in pain scores compared to the sham group. However, no statistically significant improvement was observed in quality of life between the two groups, suggesting that the beneficial effect of GTT may be specific to pain modulation rather than broader functional outcomes.

Denison (50) in a pilot clinical study explored the effects of TT on patients with fibromyalgia syndrome and reported consistent improvements in pain and quality of life. Participants experienced a statistically significant reduction in pain intensity immediately following each TT session, indicating a reproducible short-term analgesic effect. Additionally, pre-treatment pain levels progressively decreased across the course of six sessions, suggesting a cumulative benefit of the intervention. Improvements in overall quality of life were also reported at the completion of the treatment cycle. Although limited by a small sample size and the absence of a rigorous control group, the study provides preliminary evidence that TT may contribute to pain reduction and enhanced well-being in patients with fibromyalgia.

Hanely et al. (41) through a randomized, placebo-controlled trial demonstrated that both mindfulness and Reiki interventions were associated with clinically meaningful improvements in chronic knee pain. Participants receiving mindfulness or Reiki reported a significant reduction in pain intensity and functional impairment, as measured by validated knee-specific pain and function subscales, compared with the waitlist control group. These improvements were maintained at the two-month follow-up, suggesting short-term persistence of treatment effects.

Mueller et al. (49) demonstrated that TT was associated with reductions in back pain. Patients receiving TT showed a marked reduction in pain intensity, as well as a significant improvement in functional disability measured by a validated back-pain disability measure. Improvements were observed progressively over the four-day intervention period, indicating a cumulative therapeutic effect. Multivariate analysis revealed a large effect size, supporting the clinical relevance of TT as an adjunctive intervention. In contrast, patients receiving standard care alone demonstrated comparatively smaller improvements. These findings suggest that TT may enhance short-term pain relief and functional outcomes in hospitalized patients with back pain.

Wardell et al. (47) explored, through an RCT combined with qualitative analysis, the impact of HT on older adults experiencing persistent pain. Participants in the HT group received three 30-minute Healing Touch sessions per week over a two-week period. The control group, on the other hand, received presence care sessions (a form of non-intervention attention control) of similar duration and frequency, designed to control attention and interpersonal interaction. The quantitative outcome measures included pain intensity, perceived ability to cope with illness, and independence in activities of daily living. Quantitatively, no statistically significant differences were observed between the intervention and control groups in terms of pain levels or functional outcomes. However, some individuals in the HT group reported subjective improvements in pain relief and general well-being, as documented through qualitative feedback and narrative interviews. These subjective improvements included reports of enhanced relaxation, emotional release, improved mood, and better coping with pain, suggesting a potential psychosocial benefit of Healing Touch not fully captured by standard quantitative measures.

Zacaron et al. (45) investigated the effects of Laying on of Hands (LooH), both with and without a spiritual component, as a complementary therapy in patients with knee osteoarthritis (KOA). All participants underwent kinesiotherapy, while the spiritual LooH group performed kinesiotherapy combined with spiritual exercises and healing touch, and the non-spiritual LooH group received kinesiotherapy and healing touch without the spiritual component. The physiotherapy program consisted of 5 min of warm-up and stretching, 37 min of strengthening and balance exercises, and 3 min of rest. The intervention lasted 8 weeks, with a follow-up assessment conducted at 16 weeks. Outcome measures included pain and function, pain intensity, and anxiety/depressive symptoms. Results suggested that the spiritual LooH group showed statistically significant improvements compared to both the non-spiritual and control groups in pain reduction, functional improvement, and anxiety reduction.

Bicego et al. (37) evaluated and compared the effectiveness of various complementary treatments for chronic pain. The interventions included psychoeducation/Cognitive Behavioral Therapy (CBT) plus self-care, self-care only, self-hypnosis plus self-care, and music therapy plus self-care. Each group participated in seven monthly sessions, with follow-up at 6 and 12 months. The therapeutic modalities were different for each group: the psychoeducation/CBT group took part in supportive group discussions; the self-care group engaged in empowerment-based exercises centered on emotions, cognition, and behavior, rather than relaxation; the self-hypnosis group received 20-minute hetero-hypnosis exercises in addition to the self-care tasks; and the music therapy group listened to specially composed musical pieces for 20 min at the end of each session. A comprehensive battery of outcomes was assessed, including pain and fatigue intensity, anxiety/depressive symptoms, insomnia severity, pain-related disability, health locus of control, quality of life, and patient-reported global impression of change. All four groups showed significant improvement over time in pain intensity, insomnia severity, physical quality of life, and several pain-related attitudes, with no significant group effect — that is, self-hypnosis/self-care was not superior to the other three approaches. These benefits were maintained at both 6- and 12-month follow-up regardless of treatment arm.

Smith et al. (48) aimed to evaluate the effects of TT on pain, physical function, and general well-being in patients with KOA. The intervention group underwent two TT sessions per week for 8 weeks, alongside conventional physiotherapy. Assessments were conducted at baseline, at the end of the 8-week intervention, and during follow-up at 12–16 weeks post-intervention. Primary outcomes included pain intensity, functional status, and quality of life, which were investigated. The TT group showed greater improvements in pain reduction and physical functioning than the control group. Improvements were observable at the end of the intervention and were sustained during the follow-up period. While trends toward better well-being and quality of life were noted, these did not reach statistical significance.

Overall, the included studies suggest that these complementary interventions may be associated with improvements in pain perception and, in some cases, quality of life and activities of daily living.

### 3.5 Quality of the included studies

Fourteen clinical trials published between 2004 and 2025 were included in this scoping review. The included studies evaluated the reported outcomes of hypnosis-based interventions, touch-based interventions, and biofield therapies for pain management, either alone or in combination with conventional rehabilitation. Table 3 shows the clinical and descriptive data of each included study. Based on the AHRQ quality standards, the studies were rated as follow: six RCTs reached “Good quality”, four RCTs reached “Fair quality” and three RCTs reached “Poor quality”. Table 5 shows the domains that were assessed for each study. The observational study by Denison was assessed using the NOS and achieved a total score of 3/9, indicating low methodological quality and high risk of bias, due to the absence of a control group and limited outcome assessment.

**Table 5:** Cochrane risk of bias 2 (RoB 2).

Publication	Randomization	Intervention	Missing outcome data	Outcomes measurement	Results	Overall bias	AHRQ Standard
Hanely et al., (41)	Low	Low	Low	Low	Low	Low	Good
Mueller et al., (49)	Some concerns	Some concerns	Low	Some concerns	Low	Some concerns	Fair
Dorta et al., (38)	Low	Low	Low	Low	Low	Low	Good
Salgado et al.,	Low	Low	Low	Low	Low	Low	Good
Tonye-Geoffroy et al., (40)	Low	Low	Low	Low	Low	Low	Good
Bicego et al., (37)	Some concerns	High	Low	Low	Some concerns	High	Poor
Aravena et al., (36)	Low	Low	Low	Low	Low	Low	Good
Zacaron et al., (45)	Some concerns	Some concerns	Low	Low	Some concerns	Some concerns	Fair
Wardell et al., (47)	High	High	Some concerns	High	High	High	Poor
Abrahamsen et al., (35)	Low	Low	Low	Low	Low	Low	Good
Smith et al., (48)	Some concerns	Low	Some concerns	Low	Some concerns	Some concerns	Fair
Abrahamsen et al., (34)	Some concerns	Low	Low	Low	Low	Some concerns	Fair
Grøndahl et al., (39)	High	High	Some concerns	Some concerns	High	High	Poor

### 3.6 Complications

There were no reports of serious complications in any of the trials included in this review.

## 4 Discussion

This scoping review synthesized the available evidence on hypnosis-based interventions, touch-based interventions, and biofield therapies as complementary approaches to pain management and rehabilitation in physiotherapy settings. These interventions were considered together because they represent complementary, non-pharmacological approaches investigated for pain management and rehabilitation, although they differ in their theoretical basis, mode of delivery, and proposed mechanisms. Their inclusion does not imply that they share the same theoretical basis, mode of delivery, or physiological mechanism.

Overall, the included studies suggest a generally positive trend, suggesting that the included hypnosis-based interventions, touch-based interventions, and biofield therapies may contribute to reductions in pain intensity and, in some cases, improve psychological well-being, quality of life, and activities of daily living (ADL). In fact, a statistically significant difference was observed between patients who received treatment with these complementary interventions and patients who followed traditional therapy modalities. The available evidence suggests that hypnotherapy may represent a promising complementary intervention. The included studies consistently reported positive effects not only on pain reduction but also on psychological symptoms (e.g., anxiety, depression, catastrophizing) and sleep quality. Importantly, these improvements were often sustained at long-term follow-ups (6 months to 1 year), suggesting the durability of clinical benefits (35, 36, 38–40). Touch-based interventions and biofield therapies were associated with heterogeneous findings across the included studies, particularly in reducing pain intensity and improving physical function, although the evidence regarding QoL improvements was more heterogeneous (45–49). In some studies, such as Salgado et al. (46), improvements were confined mainly to pain perception, while broader QoL domains remained unaffected. Conversely, studies integrating touch with spiritual components, such as Zacaron et al. (45), suggested potential additional psychosocial benefits, suggesting that the therapeutic context may modulate treatment outcomes. Denison's (50) pilot study evaluating TT in individuals with fibromyalgia reports consistent pain reduction and improvements in QoL, suggesting potential clinical benefit, particularly in chronic, treatment-resistant populations. Studies evaluating Reiki reported improvements in pain perception, such as the clinical trial conducted by Hanley et al. (41), which demonstrated that mindfulness and Reiki treatment can improve knee chronic pain.

When synthesising across outcome domains rather than on a study-by-study basis, three patterns emerge. Pain intensity was the outcome that improved most consistently across hypnosis-based interventions, touch-based interventions, and biofield therapies. However, the strength of the evidence for this is uneven: reductions were reported in good-quality RCTs (36, 38, 40, 41, 46), as well as in smaller, poor-quality pilot studies (49, 50). This makes it difficult to estimate the overall magnitude of the effect with confidence. Psychological and quality-of-life outcomes were considerably less consistent. Improvements were reported in several hypnosis studies (35, 38, 40) and in the spiritual arm of Zacaron et al. (45). However, Salgado et al. (46) found no improvement in quality of life despite an effect on pain, Bicego et al. (37) found no difference between groups across psychosocial outcomes and trends towards improved well-being in Smith et al. (48) did not reach statistical significance. Function and activities of daily living showed a similar mixed pattern. Smith et al. (48) and Mueller et al. (49) reported significant functional gains, whereas Wardell et al. (47) found no statistically significant difference between Healing Touch and the attention control group in terms of either pain or functional outcomes. Benefits were limited to qualitative, subjectively reported improvements. This heterogeneity of effect across domains, together with the use of sham or placebo-controlled designs in several studies, suggests that contextual and expectancy effects likely contribute to the observed outcomes alongside any modality-specific mechanisms. The current evidence does not yet support the attribution of consistent clinical benefits to these interventions across all outcome domains.

A key challenge in synthesizing the evidence lies in the heterogeneity across studies. There was wide variability in terms of intervention protocols (session frequency, duration, delivery mode), control group design (placebo, sham, standard care), follow-up times, and outcome measures used. While most studies employed validated pain scales (VAS, NRS, Quebec Back Pain Disability Scale), psychological and QoL outcomes were measured with a diverse array of tools (e.g., SF-36, WHOQOL, HADS), complicating direct comparisons. Furthermore, the number of sessions varied substantially: from one or two sessions over weeks (46) to daily interventions lasting several months (36), which may impact the generalizability and reproducibility of the findings. Despite this, studies with more structured and longer interventions tended to report more sustained clinical effects, suggesting a possible dose-response relationship that warrants further exploration. Several studies compared hypnosis-based interventions, touch-based interventions, or biofield therapies with conventional treatments or active comparator conditions. These findings are consistent with the growing interest in integrative approaches that address not only the physical but also the psychological and emotional dimensions of pain. Importantly, no serious adverse events were reported in any of the included studies; however, as detailed in Table 3, adverse-event monitoring was not systematically described or instrument-based in most trials, and several studies did not report on safety at all. The available safety evidence should therefore be considered preliminary rather than confirmatory of the overall tolerability of these interventions. This non-invasive profile positions them as potentially attractive adjuncts to rehabilitation, especially for patients with chronic pain who may not fully respond to pharmacological or physiotherapeutic interventions alone, although confirmation of their safety profile in larger, systematically monitored samples is still needed.

Despite the promising findings, the overall quality of the evidence remains variable. According to AHRQ standards, only six of the thirteen RCTs were rated as high-quality, with the remaining studies classified as fair or poor due to methodological limitations such as small sample sizes, lack of blinding, unclear randomization processes, or incomplete outcome data.

Notably, positive findings across the included intervention categories are supported by a mix of evidence rather than uniformly strong evidence. Among hypnosis-based studies, several good-quality RCTs report consistent benefits (36, 38, 40), but two of the most favourable reports (37, 39) derive from poor-quality, small or non-superior trials. Among studies evaluating touch-based interventions and biofield therapies, good-quality evidence (41, 46) coexists with fair- or poor-quality pilot studies (47–50) reporting smaller or mixed effects. Specifically, Wardell et al. found no statistically significant quantitative difference between groups.

Another limitation concerns publication bias: although no formal assessment (e.g., funnel plot or grey literature search) was conducted, restricting this review to peer-reviewed, database-indexed sources may have limited the detection of unpublished negative or null findings, and this possibility should be considered when interpreting the overall direction of the evidence. The search strategy for this review was intentionally limited to studies reporting quality-of-life outcomes alongside pain, in line with the specific aim. This approach may have excluded hypnosis trials that reported on pain outcomes without including quality-of-life measures. Modality-specific systematic reviews would be required to capture the broader literature.

Future research should focus on conducting high-quality, multicenter RCTs with standardized intervention protocols and longer follow-ups to confirm the durability of therapeutic effects. Studies should also aim to better characterize patient subgroups that are most likely to benefit from these interventions. Moreover, mechanistic studies are needed to elucidate the neurobiological and psychosocial pathways potentially associated with hypnosis-based interventions, touch-based interventions, and biofield therapies. The integration of patient-reported outcomes and qualitative methodologies, as seen in Wardell et al. (47), may also help to capture benefits not reflected in traditional scales, especially in the domains of emotional processing and subjective well-being.

## 5 Conclusion

This scoping review summarizes the emerging evidence on hypnosis-based interventions, touch-based interventions, and biofield therapies as complementary approaches to pain management and rehabilitation. The included studies reported potential improvements in pain intensity and, in some cases, psychological well-being, quality of life, and functional outcomes; however, the consistency and strength of these findings varied across intervention categories. Despite methodological heterogeneity and variable study quality, the overall findings tentatively support considering these non-pharmacological, low-risk interventions as adjunctive options alongside standard care, especially in the management of chronic pain, although the small sample sizes, heterogeneous protocols, and limited blinding in several included studies warrant caution in interpreting their clinical effectiveness. Given their generally non-invasive delivery, hypnosis-based interventions, touch-based interventions, and biofield therapies may be considered adjunctive rather than substitutive approaches within person-centred rehabilitation pathways. Safety monitoring was inconsistently reported across the included studies. Therefore, further high-quality research is needed to confirm these results, optimize treatment protocols, and clarify the mechanisms through which these therapies exert their effects. Standardized methodologies and long-term follow-up studies will be essential to fully understand their therapeutic potential and inform clinical guidelines. Larger, multicenter randomized controlled trials with standardized intervention protocols are still needed before more definitive clinical recommendations can be made.

## Data Availability

The original contributions presented in the study are included in the article/Supplementary Material, further inquiries can be directed to the corresponding author.
